# General Practitioners’ and patients’ perceptions towards stratified care: a theory informed investigation

**DOI:** 10.1186/s12875-016-0511-2

**Published:** 2016-08-31

**Authors:** Benjamin Saunders, Bernadette Bartlam, Nadine E. Foster, Jonathan C. Hill, Vince Cooper, Joanne Protheroe

**Affiliations:** Arthritis Research UK Primary Care Centre, Research Institute for Primary Care & Health Sciences, Keele University, Keele, Staffordshire UK

**Keywords:** Stratified primary care, General practice, Behaviour change interventions, Musculoskeletal conditions, Qualitative, Interviews, Focus groups, Theoretical Domains Framework (TDF)

## Abstract

**Background:**

Stratified primary care involves changing General Practitioners’ (GPs) clinical behaviour in treating patients, away from the current stepped care approach to instead identifying early treatment options that are matched to patients’ risk of persistent disabling pain. This article explores the perspectives of UK-based GPs and patients about a prognostic stratified care model being developed for patients with the five most common primary care musculoskeletal pain presentations. The focus was on views about acceptability, and anticipated barriers and facilitators to the use of stratified care in routine practice.

**Methods:**

Four focus groups and six semi-structured telephone interviews were conducted with GPs (*n* = 23), and three focus groups with patients (*n* = 20). Data were analysed thematically; and identified themes examined in relation to the Theoretical Domains Framework (TDF), which facilitates comprehensive identification of behaviour change determinants. A critical approach was taken in using the TDF, examining the nuanced interrelationships between theoretical domains.

**Results:**

Four key themes were identified: Acceptability of clinical decision-making guided by stratified care; impact on the therapeutic relationship; embedding a prognostic approach within a biomedical model; and practical issues in using stratified care. Whilst within each theme specific findings are reported, common across themes was the identified relationships between the theoretical domains of *knowledge*, *skills, professional role and identity, environmental context and resources,* and *goals*. Through analysis of these identified relationships it was found that, for GPs and patients to perceive stratified care as being acceptable, it must be seen to enhance GPs’ *knowledge* and *skills*, not undermine GPs’ and patients’ respective *identities* and be integrated within the *environmental context* of the consultation with minimal disruption.

**Conclusions:**

Findings highlight the importance of taking into account the context of general practice when intervening to support GPs to make changes to their clinical behaviour. Findings will inform further stages of the research programme; specifically, the intervention format and content of support packages for GPs participating in a future randomised controlled trial (RCT). This study also contributes to the theoretical debate on how best to encourage clinical behaviour change in general practice, and the possible role of the TDF in that process.

**Electronic supplementary material:**

The online version of this article (doi:10.1186/s12875-016-0511-2) contains supplementary material, which is available to authorized users.

## Background

### Stratified care for musculoskeletal conditions

Musculoskeletal conditions represent a considerable worldwide healthcare burden and, in the UK, account for 14 % of all general practice consultations [1]. Whilst usual care commonly follows a ‘stepped’ approach, with patients initially given low-intensity treatments, moving onto subsequent levels of treatment if interventions at each step fail, stratified care involves ‘targeting treatment to patient subgroups based on key characteristics such as their prognostic profile, likely response to specific treatment and suspected underlying causal mechanisms’ [2]. It aims to ‘identify those who will have the most clinical benefit or least harm from a specific treatment’ in order to ‘make the best decisions for groups of similar patients’ [3]. Prognostic stratified primary care, as is the focus of the larger research programme of which this paper is part, involves completing a brief self-report tool to identify patients’ risk of persistent disabling pain and matches patient subgroups to appropriate early treatment options [4]. As a result, patients needing more intensive treatment are identified at the earliest possible stage, allowing them to be ‘fast-tracked’ to appropriate services, whilst patients with a good prognosis can be reassured and unnecessary treatments avoided. The move from a stepped care model to a stratified approach clearly requires a change in GP behaviour; which includes shifting from a predominantly biomedical approach, common in current usual practice and often centring on diagnosis [5], to integrating information about prognostic factors that include psychosocial obstacles to recovery; an approach in line with a biopsychosocial model of care [6].

Whilst management of musculoskeletal conditions using a stepped care approach adheres to current UK guidelines for low back, neck, knee and shoulder pain from The National Institute for Health and Care Excellence (NICE) [7], for patients with low back pain evidence suggests that stratified primary care results in improved health outcomes and cost savings when compared with best current care and usual primary care [4, 8]. Given findings that similar prognostic factors predict outcome across different body region pain sites [9], the Stratified Primary Care for Musculoskeletal Pain five year research programme (2014–19) is developing and testing a new stratified care intervention for patients with the five most common musculoskeletal pain presentations in primary care – back, neck, shoulder, knee and multisite pain. The prognostic tool being developed will stratify patients into one of three subgroups: low, medium or high in relation to risk of persistent disabling pain. Matched treatment options are recommended for each subgroup. It is intended that completing the tool will take no longer than two minutes within the consultation. Later stages of the programme involve a large randomised controlled trial (RCT) to test the clinical and cost-effectiveness of stratified primary care versus usual care.

As part of this programme, this paper reports on qualitative research aiming to investigate the views of patients and General Practitioners (GPs) about the acceptability of prognostic stratified primary care for adults with musculoskeletal pain conditions, and the anticipated barriers and facilitators to its use in clinical practice. Findings will be used both to refine the stratified care intervention and support its use within the RCT. Specifically, data-analysis will identify key determinants of behaviour change to be targeted in supporting GPs to deliver stratified care; which will inform both the design of the format in which the stratified care tool will be presented, as well as the design and content of support packages for GPs participating in the RCT.

### The Theoretical Domains Framework (TDF)

Changing healthcare practice holds diverse challenges [10, 11], including whether the intervention is considered meaningful and relevant, and the degree to which clinicians prefer working within established frameworks [12], as well as perceived threats to clinical autonomy [13]. Additionally, practical constraints around the consultation are also key, not least time-constraints in general practice [10]. Examining such challenges using a theoretically-underpinned approach can extend the scope of purely descriptive approaches, enabling a more cogent and coherent explanation of the issues identified in the data. In this research we used the Theoretical Domains Framework (TDF), a model that has gained increasing popularity in the design and implementation of complex interventions. Developed by Michie et al. [14] and further refined by Cane et al. [15], the TDF synthesises 112 psychological constructs determining behaviour change into fourteen domains, which can be used to identify barriers and facilitators to behaviour change in the context of clinical interventions. These theoretical domains include: knowledge, skills, social/professional role and identity, and memory, attention and decision-processes (see: [15]). Considering change at the level of the individual, the TDF also captures broader organisational and cultural factors, e.g. environmental context and resources, as well as social, economic and policy influences, providing a comprehensive framework for intervention design and evaluation. The TDF has been used in a range of clinical settings, including the treatment of osteoarthritis [11], mild traumatic brain injury [16], diagnosis and management of dementia [10], HPV infection, vaccination, and testing [17], upper respiratory tract infection [18], pre-operative testing [19]; acute low back pain [20]; and prescribing errors [21].

The TDF does not identify possible relationships between domains but instead presents behaviour change determinants as discrete entities, which fails to account for the complexity of everyday medical practice. Duncan et al. [21] attempted to address this limitation through identifying interrelationships between domains in relation to their data. Building on the positive steps taken by Duncan et al., we adopt a critical approach through an analysis of the relationships between theoretical domains identified within the key themes emerging from the data; highlighting which domains appear to relate to one another, how domains may be seen to interrelate, and how domains manifest within participants’ talk. Discussion of the findings is situated within the wider healthcare intervention literature and social science literature; it is intended that findings can contribute to this research literature from both an empirical and theoretical perspective.

## Methods

### Data-collection

Data were collected through focus groups and interviews, enabling in-depth exploration of the GPs’ and patients’ views. Four focus groups and six one-on-one telephone interviews were conducted with GPs (*n* = 23), and three focus groups with patients (*n* = 20), between September 2014─January 2015. GPs were recruited through practices located in the West Midlands of England that had participated in an earlier observational cohort study (the Keele Aches and Pains Study (KAPS) [22]), as well as through known clinical networks. Patients were recruited via phone and then in writing, having consented to further contact as part of the earlier cohort study. Patients were purposively sampled to obtain a sample with diverse characteristics; including, age, socioeconomic status and reported pain site/severity. Although not sampled for particular characteristics, GPs represented a range of experience levels and length of time in practice, as well as varying degrees of familiarity with a prognostic approach to stratified care for low back pain [4]. GP focus groups were held in GP practices; one of the patient groups took place at a local GP practice and the other two at a community centre. Focus groups and interviews lasted between 27 min and 1 h 19 min. All participants were given an information letter explaining the study prior to providing written informed consent. All focus groups and interviews were audio-recorded and transcribed in full. All transcriptions were anonymised.

Focus groups were facilitated by two members of the research team, both from a social science, qualitative research background. Telephone interviews were carried out by one of these two researchers with those GPs unable to attend the focus groups, with the aim to further explore the findings emerging from the groups. Before the start of each focus group/ interview, the researchers gave an informal 5–10 min presentation explaining the principles and background to prognostic stratified care for musculoskeletal conditions. The presentation given to patients was a less technical, lay version of that delivered to GPs, avoiding clinical terminology. Given that the prognostic tool and matched treatment options were still being developed, views were sought on the principles of stratified care more generally, as opposed to the specifics of the tool items and treatment options; though the existing widely used stratified care tool for low back pain: the STarT Back tool [4], was shown to exemplify the stratified care approach.

Topic guides were used, which focused on the acceptability and use of stratified care in clinical practice. The guides functioned as an aide memoire for the researchers, and not as a structured list of questions and probes. Reference to the TDF domain labels were included to remind the researchers to probe domain-related issues if they emerged within focus groups/interviews, but given the inductive aims, topic guides did not include specific questions around TDF domains. Both researchers were experienced in qualitative data-collection and in using the TDF; therefore, these minimal prompts were sufficient to allow for probing domain-relevant responses. Guides were revised iteratively on the basis of emergent findings (see Additional files 1 and 2).

### Analysis

Analysis was an iterative process and data collection continued until data-saturation was reached, with no new themes emerging. A two stage framework was adopted incorporating an inductive thematic analysis followed by a deductive process using the TDF. Anonymised transcripts were systematically coded on a line-by-line basis by one of the authors (BS) with the aid of the software program Nvivo 10, in order to identify recurrent concepts inductively. This reflected a desire to avoid imposing meaning upon the data, or potentially missing important findings which may have been risked if data were coded deductively using the TDF domains. Coding was reflexive and recursive, with codes being revisited in light of the findings of subsequent data-collection.

A sample of five transcripts was independently coded by two other authors (BB and JP) to check for inter-coder reliability. Coders brought different disciplinary perspectives to the data (BS, medical sociology; BB, social gerontology; JP, academic general practice), and the aim of independent coding was therefore to understand cross-disciplinary perspectives on the data and, through discussion, to come to an agreement on shared meanings and interpretations. For this reason it was deemed too simplistic to statistically calculate levels of agreement as a means of assessing reliability, and this was instead achieved in a more nuanced manner through detailed discussion.

Analysis of the data used the constant comparative method [23], looking for connections within and across focus groups and interviews, and across codes, highlighting data consistencies and variation. Analysis began with GP data and then mapped the views of patients against that, in order to allow for direct comparison between the two. Through this analysis four key, higher-order themes were identified, with several subthemes in each.

The second stage of the analysis involved mapping the TDF domains onto the identified themes to explore which domains could be seen to relate to these themes. A high level of ‘fit’ was observed, with more than one domain interpreted as relevant to each key theme. Beyond this, analysis explored how the TDF domains could be seen to relate to one another within the identified themes. This involved examining each coded data-extract in relation to the identified domains in order to interpret how the domains manifested in the participants’ talk and how domains could be seen to interrelate.

This analysis allowed for an appreciation of the complexity of the qualitative data, in turn reflecting the complex nature of everyday general practice; demonstrating that determinants of an individual’s reported views and behaviours are rarely discrete, and instead these influences may be interrelated. In what follows we report the findings on the four key themes and the analysis of these themes in relation to the TDF.

The study received ethical approval from the NHS REC 01 South East Scotland (Ref: 14/SS/0083).

## Results

### Participant characteristics

Patients were aged 22–85 years (mean age 58); had reported pain duration ranging from less than one week to over one year, and reported pain at one or more of the five pain sites (back, neck, shoulder, knee or multisite pain). Seven patients were male and 13 female, and represented a range of occupational backgrounds (see Appendix for a full outline of patient characteristics). Nine GPs were female and 14 male, and ranged from newly qualified clinicians to >20 years as a general practitioner. Most reported being aware of, but having limited knowledge about, a stratified care approach for treating low back pain. However, five of the GPs had used STarT Back in routine practice.

### Principal findings

The key themes and sub-themes identified through the thematic analysis are as follows:

1. Acceptability of clinical decision-making guided by stratified care (subthemes: understanding of stratified care; GP clinical autonomy).
2. Impact on the therapeutic relationship (subthemes: patient choice; conflict or collaboration).
3. Embedding a prognostic approach within a biomedical model (subthemes: fear of missing serious pathology; lack of confidence in treating patients with musculoskeletal pain).
4. Practical issues in using stratified care (subthemes: time-constraints of the consultation; resources).

### Acceptability of clinical decision-making guided by stratified care

GPs were receptive to the principles of stratifying patients, and felt that subgrouping musculoskeletal pain patients using the prognostic tool could help decision-making, when supplemented by their own judgement and experience:

Instead of thinking ‘what do I think would work here?’ I would primarily look at the tool but I would still retain that personal judgement ‘well, hang on a minute, in this case I’m not so sure’. So I wouldn’t want to say ‘right, I’m going to use the tool blindly’ but I think it would be a significant decision aid and I think I’d need a reason to deviate rather than a reason to follow.

(Male GP; Interview 6)

As well as the reported acceptability of the prognostic tool, there was evidence that GPs felt that having matched treatment options recommended to them was acceptable:

I’d be happy with it saying, ‘This is the most appropriate treatment,’ because that’s where you want to go. And more often than not you’ll think, ‘Yeah, that’s more or less what I had in mind anyway.‘ And that’s fine. Or you’ll go, ‘Oh, that’s a good idea. That wasn’t quite what I was going to do but it might make sense’, in which case, fine.

(Female GP 2; Focus Group 4)

This GP expresses openness to making treatment decisions in line with the recommended options, which it is felt would in any case usually correspond with decisions made independently of using stratified care. She does, however, also express a willingness to follow treatment recommendations which may differ to those she would have ordinarily chosen, as long as these clinically ‘make sense’; therefore, from this perspective, availability of matched treatments does not represent an intrusion upon usual clinical decision-making.

The view that stratified care can be an acceptable addition to, rather than a replacement for, existing clinical judgement in making treatment decisions is further emphasised in the following extract:

An awful lot of medicine is an art and that’s hopefully where we come into it but there’s an awful lot of evidence of what we should be doing and why, and that’s why we don’t find tools and protocols threatening. I know some surgeries do. Some surgeries are very anti: ‘it’s taking away all your decision-making’. Well, actually we’ve got millions of decisions to make and we don’t always get them right, so anything that helps us do that makes sense to me.

(Male GP 1; Focus Group 2)

Evidence was also apparent that patients perceive stratified care as being acceptable; both in terms of the positive view of being placed on a particular treatment pathway, and recognition of the value of patients receiving appropriate management as a result of stratification:

I think that would give people a sense of satisfaction to know that the doctor’s going to do a bit of a pathway for you, personally… And if it’s guiding the right people to physio and the right people to self-help or medication, then I think it’s got potential.

(Female Patient 2; Focus Group 1)

When examined in relation to the TDF, these findings were identified as having particular relevance to the theoretical domains of *knowledge*, *skills* and *professional role and identity.* These GPs and patients displayed positive *beliefs about the consequences* of using stratified care with regard to enhancing the GP’s existing *knowledge* and *skills* in the assessment and management of musculoskeletal pain patients; but also saw clinical judgement as retaining an important role, thus a key element of the GP’s *professional role and identity* can be maintained.

A contrasting position, however, was of stratified care not adding significantly to GP decision-making, or to their clinical judgement, and of it potentially leading to reduced clinical autonomy; a view which, amongst many GPs, appeared to reflect broader concerns about the increase in use of clinical ‘tick-box’ tools in general practice; indicating a lack of acceptability of a stratified care approach:

I am not sure that they actually add anything [i.e. decision aids or tools]. I think they provide a very simplistic way of dealing with very complex problems which I’m sure for some people is advantageous, but I’m not convinced…the GP being a diagnostician and having some sort of nous about things, and I think it takes that away, if you’re just ticking boxes and working through a piece of paper. I think these problems and patients are much more complex than that.

(Male GP; Interview 1)

The view was also expressed that treatment decisions, particularly regarding onward referral to physiotherapy, were unlikely to be influenced by the recommended matched treatment options; indicating that some GPs did not see added value in the approach:

I think we use our physios quite often, quite appropriately, I’d be surprised if I referred less to a physio from using your approach. So I don’t think it would change as a result of these options being recommended.

(Male GP 2; Focus Group 2)

Patients, too, stressed the importance of GPs’ clinical judgement and experience in informing treatment decisions, expressing a concern about the potential for some GPs to rely solely on the results of a brief prognostic tool:

There is a tendency amongst certain doctors to just rely on numbers and statistical analysis rather than actually dealing with the patient. And so they just go, ‘Oh, you’re a number six so you’re having this treatment’…And if a patient comes out with a low score but you feel from speaking to them, and what you know of them, that it is actually a high risk, I would rather the doctor use their experience and their skills as a General Practitioner in actually dealing with patients coming in rather than just relying on the numbers.

(Male Patient 1; Focus Group 1)

The views reflected in these three extracts indicate a different manifestation in theoretical domains; with the perception that stratified care would not significantly or meaningfully add to the GP’s existing *knowledge* and *skills*, and could undermine their *professional role and identity* because of reduced clinical autonomy.

**Fig. 1:**
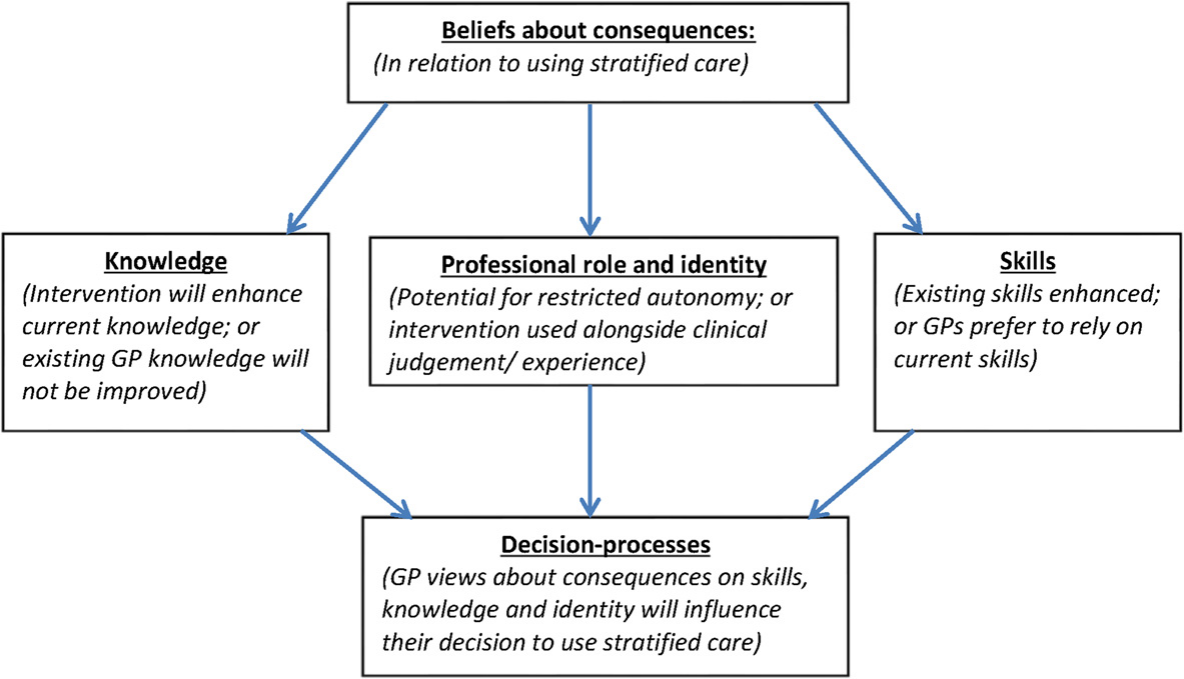
GP and patient perceptions of stratified care regarding decision-making. Represents diagrammatically the identified relationship between the theoretical domains as explained above, with ‘beliefs about consequences’ in relation to stratified care shown to relate unidirectionally to the domains of ‘knowledge’, ‘skills’ and ‘professional role and identity’; which are in turn shown to relate to the domain of ‘decision processes’

Taking into account the variation in the data within this theme, a relationship between theoretical domains may be proposed whereby GPs’ *beliefs about consequences* of using stratified care, in relation to their *knowledge, skills* and *professional role and identity*, may in turn affect their *decision processes* with regard to how stratified care informs clinical decision-making; as demonstrated in Fig. 1.

### Impact on the therapeutic relationship

Some GPs felt that using the prognostic stratified care approach could enhance the therapeutic relationship by facilitating greater dialogue, and that patients would respond positively to the GP investing more time in their problem:

They [patients] are used to us asking them to do the questions, filling in all sorts of things and knowing that it’s…something to help us. Just the same as patients are reassured when we look things up in a book; they’re not thinking we’re crap, they’re thinking actually ‘look at the time he’s taking to do that’. So I don’t think I’d have any problems saying ‘there is this tool, it’s very simple, is it okay if we use that and ask some questions and that might help us to come to a decision?’

(Male GP 1; Focus Group 2)

The view that the use of stratified care may lead patients to feel positive about GPs appearing to show more care and interest in their problem was also evident in the patient focus groups, as these two male participants discuss:

Patient 1: Just the fact that they [patients] feel the doctors are interested in them and the fact that-

Patient 2: The fact that somebody cares.

Patient 1: Exactly.

Patient 2: The fact that somebody cares enough to ask you questions.

(Focus Group 1)

When examined in relation to the TDF, these findings point to a relationship between the domains of *beliefs about consequences, goals* and *professional role and identity*. The positive beliefs about the consequences of using stratified care with regard to the goals of facilitating discussion and patient engagement can also be seen to positively relate to the respective roles and identities of the GP and patient, with both seen as active participants involved in a dialogic relationship, potentially leading to greater patient satisfaction.

A contrasting view was also presented by GPs, however, that stratified care could impact negatively upon the therapeutic relationship. One concern was that completion of the prognostic tool could impede upon the GP’s efforts to build rapport with the patient:

Tools like this can break down the consultations at times…You find that you’re talking to a patient and you’ve got a really good rapport with them and suddenly you say, ‘Right,’ and then you go to these questions which, if you’re used to asking things in a certain way, it’s difficult then to resort to someone else’s way of saying something.

(Female GP 3; Focus Group 3)

GPs also anticipated a potential for conflict if the matched treatment options were not in line with patients’ treatment preferences. Some expressed concern about patient choice being restricted to particular matched treatment options. As a result, these GPs were anxious about potential difficulty of enabling patients to see the appropriateness of matched treatments; especially referral to services with long waiting times. This led some to imply an adversarial relationship:

It’s [stratified care] using resources in the most clinically appropriate way. But in doing so you do risk, if you’ve got very specific treatments, you’ve got an injection, you’ve got physio, you’ve got to wait eight weeks, then people are going to have their own feelings about that… So my worry about it is that it sort of dupes the patient decision… it’s like a trump card, almost. If the system is set up so that patients have got to- if they score 10, then they go here, and if they score 5, they go there but, ‘well I don’t want that.’ Then who wins?

(Female GP 2, Focus Group 3)

The potential for conflict between GPs and patients as a result of stratified care was echoed by patients, who expressed concerns about being classified into a subgroup that they may not feel is appropriate to their pain problem; and subsequently that the recommended matched treatments would not correspond to their treatment preferences:

My fear would be that if you were put on Level 1 care [low risk] and…you’re saying to the doctor ‘Look, the pain is here, it’s doing this, I can’t do this, it’s depressing me, I’m scared what’s going to happen with this’, and he disagrees and keeps you on Level 1. You know, it’s an argument with the doctor.

(Male patient 1, Focus Group 2)

**Fig. 2:**
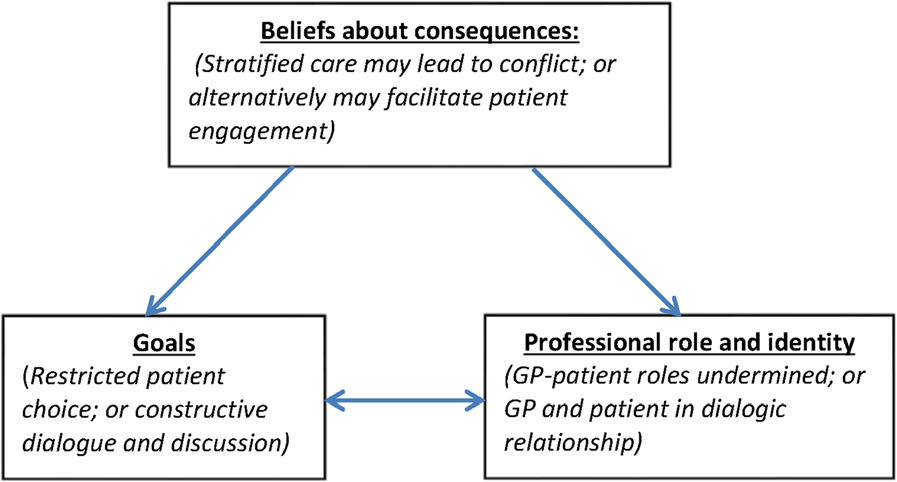
Perceived impact of stratified care on the therapeutic relationship. Represents diagrammatically the identified relationship between the theoretical domains as explained above, with ‘beliefs about consequences’ shown to relate unidirectionally to the domains of ‘goals’ and ‘professional role and identity’; which are also represented as relating to one another

For these GP and patient participants, their *beliefs about consequences* led to a concern that stratified care might hinder the *goal* of establishing an effective therapeutic relationship between the GP and patient. Threats to this relationship were seen as having the potential to undermine key elements of the GP’s and patient’s respective *roles and identities*. The relationship between theoretical domains with regard to the variation described within this theme can be represented as follows: Fig. 2.

### Embedding a prognostic approach within a biomedical model

GPs reported being inclined to stay within a strict biomedical frame when a patient first presents with musculoskeletal pain, with possible psychosocial factors related to the condition only explored if the patient re-consults about the problem several times. This biomedical focus appears to relate to the GP’s need to prioritise within the strict time-constraints of the consultation, meaning that after the primary objectives of history-taking, examination and consideration of possible serious pathology, there is little time for additional tasks; and prognostic factors are rarely a key focus. The reference in the following extract to general practice and medicine more widely suggests that the participant sees this as being part of the organisational culture of the discipline:

General practice and medicine is based around taking the history and doing an examination. And taking the history certainly we tend to focus initially on how the pain is affecting the patient… I don’t tend to delve too deeply into the more psychosocial aspects of how their pain is affecting them. So taking them generally at face value in the first instance… rather than necessarily delving too deeply into the background.

(Male GP 1; Focus Group 4)

Some participants argued that a key role of the GP is as a diagnostician: ‘*There’s three things important in medicine. One is diagnosis, two is diagnosis, three is diagnosis’.* (Male GP 1, Focus Group 1)*.* Related to this was a concern that overreliance on the stratified care tool and matched treatment options may result in GPs becoming less proficient in diagnosing musculoskeletal conditions:

Do you risk de-skilling GPs and their clinical acumen? Because you need to be able to diagnose particular conditions - your biceps tendon rupture or you’ve dislocated… those things.

(Female GP 2; Focus Group 3)

Views about the dominance of the biomedical approach and primary importance of diagnosis were shared by patients, who commonly argued that a diagnostic scan was the most effective route to resolution of their symptoms. It may therefore be the case that patient expectations of receiving a specific diagnosis further influences some GPs to focus only on the traditional biomedical approach to practice:

This is very general [the stratified care tool] because really the only way to really know what’s going on is to give somebody a scan…the only way really to sort the treatment out I think is a scan.

(Female patient 1, Focus Group 3)

The predominance of the biomedical approach amongst GPs was reportedly driven not only by cultural norms, but fear of missing serious underlying pathology that may have severe implications for the patient; a fear, it was suggested, more prevalent when examining some pain sites than others and often associated with a lack of confidence around diagnosis of musculoskeletal conditions:

There is a fear there. If you’re going to miss a shoulder problem it potentially isn’t as life limiting as if you miss a back problem. So there is a different fear element from the doctor’s perspective as well as maybe the patient’s perspective. And different sites have different fears associated with them.

(Female GP 3, Focus Group 3)

For some, part of this fear of overlooking serious underlying pathology related to being held accountable:

The problem is that if you do miss something, you get blamed. And you don’t want to be blamed… [this preoccupation] far outweighs proper medicine and logical thought really’.

(Male GP, Interview 2)

**Fig. 3:**
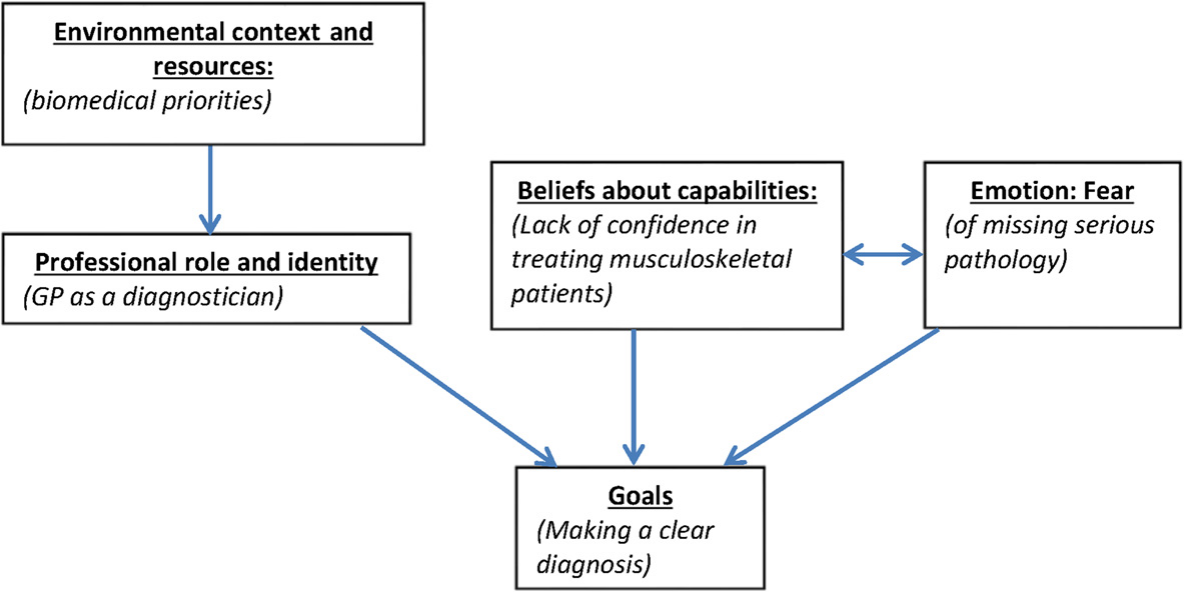
Factors influencing GPs’ orientation to a primarily biomedical approach. Represents diagrammatically the identified relationship between the theoretical domains as explained above, with ‘environmental context and resources’ shown to relate unidirectionally to ‘professional role and identity’, which in turn relates unidirectionally to the domain of ‘goals’. Additionally ‘beliefs about capabilities’ and ‘emotions: fear’ are shown to mutually relate to one another and to both individually relate to ‘goals’

These findings were identified as relating to several TDF domains. The impact of the *environmental context* of general practice, seen by GPs as supporting a biomedical focus, may impact upon their *professional role and identity*, by leading some to view their primary role as being a diagnostician. This coupled with a lack of confidence regarding their *beliefs about capabilities*, and the domain of *emotions: fear* may lead to the perceived *goals* of the consultation as being centred on making a diagnosis; which could present a barrier to the adoption of prognostic stratified care. This relationship between domains is represented in Fig. 3, below:

However, some GP participants placed less emphasis on diagnosis, making a distinction between the routine task of assessing for serious pathology, and the less routine outcome of making a concrete diagnosis. As a result they saw added value in stratified care through being able to provide patients with prognostic information in the face of diagnostic uncertainty:

Male GP 2: I think in general practice you’ve got to feel comfortable about not making a diagnosis. [Female GP 1: Yeah] There’re not many times you do, to be honest… You think it might be that but you’re not sure, it could be this other. You’re not actually making a diagnosis, you’re just doing this with your screening procedure, screening for sinister things or the serious things.…

Female GP 1: No. And the other thing as well is being able to say to patients ‘I don’t know what it is, but I know what it isn’t’.

(Focus Group 1)

### Practical issues in using stratified care

Regarding the use of stratified care in routine practice, some GPs expressed concerns that completing even a short tool could detract from salient elements of the consultation and disrupt its flow and structure, resulting in an altered style of consulting:

You’re assessing the patient, doing whatever is necessary for the patient, *then* you have to do the questionnaire. The consultation is then in two stages. You’ve got the consultation as I understand it and then you’ve got the questionnaire-filling part of the consultation, which somehow, in my mind, is different…you have a doctor sitting there, talking to a patient and getting yes/no answers. I like it to be a two-way street, whereas a questionnaire changes that.

(Female GP 1; Focus Group 4)

Concerns were also expressed about the time needed to use the approach. It was emphasised that stratified care would only be seen to be acceptable to GPs if it is quick to complete:

It’s got to be very, very quick and slick because a couple of minutes doesn’t sound much but if you’re working a full day and you’re dealing with 40, 50, sometimes 60 patients, including a few on the phone, if you run over by five minutes by each patient, that’s five hours.

(Male GP 1; Focus Group 3)

Issues relating to the time-constraints of the consultation were also raised by patients, leading to some scepticism about whether the treatment model, and in particular the prognostic tool, would be used in practice:

Male patient 2: Are GPs really going to have the time to do this? The surgery I go to, you get about four minutes, five minutes consultation. Are they really going to let you sit there and fill a form in?

Female patient 1: I think you’re right.

Male patient 2: I don’t think it’s going to happen.

(Focus Group 1)

Another condition highlighted by GPs as being necessary in order for stratified care to be perceived to be acceptable is that the recommended matched treatments must correspond to locally-available onward referral services:

Whatever decision you come out with, the care option that it’s recommending must be embedded within the healthcare system that you’re working in. So there’s no point of having these treatment options in a tool if it’s not going to be available in your practice area.

(Female GP 2; Focus Group 3)

In relation to the TDF, again the domain of *environmental context and resources* is particularly relevant to this theme; as well as *goals* and *pessimism*. GPs’ and patients’ concerns that aspects of the contextual environment of the consultation such as time-constraints could be a barrier to the use of stratified care, as well as that use of the tool could undermine priority *goals* and disrupt the consultation structure, resulted in *pessimism* about its use in practice. Concerns over the availability of services for onward referral also indicate a potential barrier relating to the domain of *environmental context and resources*, regarding the broader context beyond the consultation itself.

However, some GPs took a different position, identifying past experience of routine use of similar tools for other conditions (e.g. for depression, dementia, fracture risk) as an enabler to the use of stratified care; and there was evidence of the view that if it proves clinically useful the approach would be a welcome addition to consultations:

I think we’re pretty used to anything that can improve outcomes and quality and help us make the decisions, I’d be keen to use…I think if the tool is validated and also secondary care and the other services you’re referring to understand that the tool is useful and perhaps have some background to it as well, then I can’t see it being anything but useful.

(Male GP 1; Focus Group 2)

**Fig. 4:**
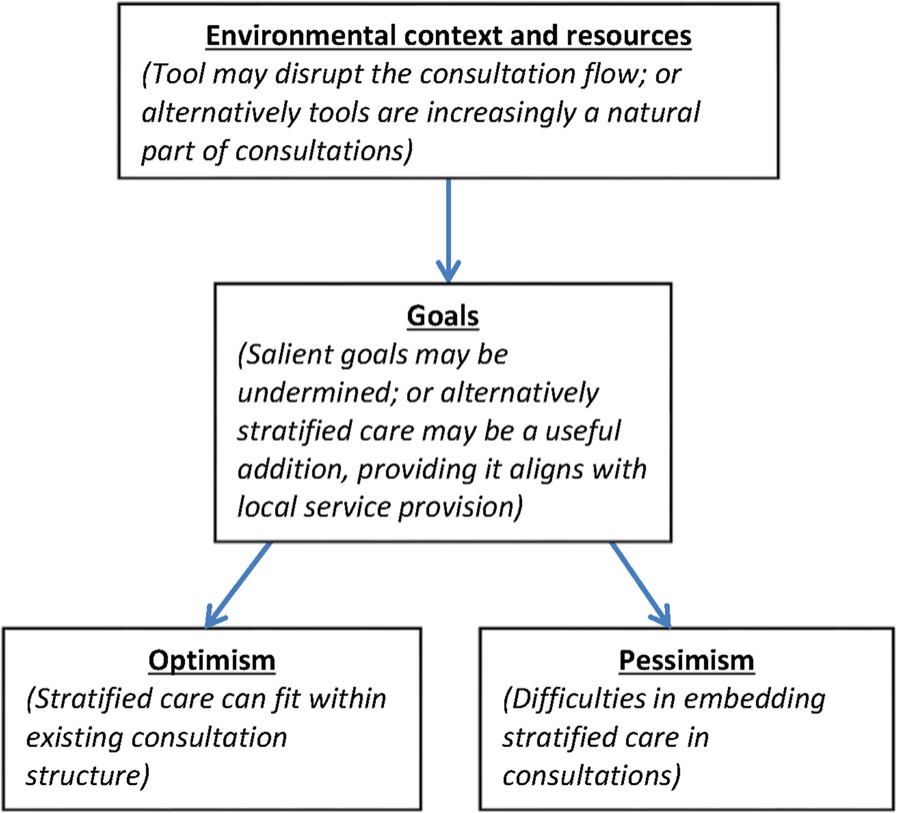
Perceived impact of stratified care on routine consultations. Represents diagrammatically the identified relationship between the theoretical domains as explained above, with ‘environmental context and resources’ shown to relate to ‘goals’, which is in turn displayed as relating unidirectionally to both ‘optimism’ and ‘pessimism’

From this perspective, stratified care was not perceived as necessarily impacting negatively on the environmental context of the consultation, and was instead seen as as potentially benefitting practice, leading to *optimism* about its use; again, however, on the proviso that it links with onward referral services. The relationship between domains with regard to these differing views can be displayed as such: see Fig. 4.

## Discussion

In this article we have presented the perspectives of GPs and patients on the acceptability, and anticipated barriers and facilitators to the use stratified care for the five most common musculoskeletal conditions, with both similarities and differences in views reported across identified themes, which were analysed in relation to the TDF.

A key finding within the first theme – acceptability of clinical decision-making guided by stratified care – related to how the approach is perceived to impact upon the theoretical domains of *skills*, *knowledge* and *professional role and identity*, and how this in turn may inform the GP’s *decision processes*. A salient concern of some GPs was that the approach could undermine the GP’s professional identity by leading to reduced clinical autonomy. This concern is consistent with literature on clinicians’ desire to maintain independence from external influence on their clinical work (e.g. [24, 25]). As Powell and Davies ([26], p.808) contend, ‘a key facet of professional identity for doctors is the desire to practise as autonomous individuals who retain personal control over how they define, sequence and evaluate their work’. In our data, the desire for clinical autonomy was commonly discussed in generalised terms, not specifically relating to the anticipated impact of stratified care, appearing therefore to indicate a general resistance by some GPs to the rise of what Flynn [27] refers to as the ‘prioritisation of codified knowledge’ – that is, clinician knowledge becoming increasingly systematised, with tacit, experience-based knowledge gradually being undermined. Whilst stratified care is not intended to act to ration access to healthcare, it was clear that some GPs homogenise all such clinical tools in terms of their perceived negative effect on clinical autonomy. Concern over the reduced role of clinical experience was also reflected in the patient data, perhaps supporting Lupton’s [28] argument that despite a contemporary trend that sees clinicians now more open to question, an enduring respect for the GP’s clinical judgement remains strong amongst many patients.

In contrast, the contributions of some GPs reflected a different manifestation in theoretical domains – they perceived their existing knowledge and skills in the assessment and management of musculoskeletal pain patients as potentially enhanced by stratified care, suggesting a positive impact of codified knowledge. Therefore, whilst much of the existing research literature focuses on clinicians striving for autonomy, this finding aligns with recent studies reporting both positive and negative clinician views towards clinical decision-aids [29], including a study of the acceptability of stratified care for low back pain in Germany [30]. Such findings could indicate a shift on the part of some clinicians towards becoming increasingly accustomed to, and accepting of, the use of clinical tools to support decision-making; a view also reflected by some patients who perceived stratified care as an acceptable addition to their usual treatment, showing recognition of its potential added value in informing treatment decisions.

Importantly, however, the reported views of those GPs who perceived stratified care as an acceptable addition to practice did not suggest the abandonment of autonomy, but autonomy being exercised alongside, or within the frame of, the use of stratified care; helping rather than replacing good clinical judgement. This lends support to Evetts’s [31] proposition that ‘discretion’ may be a more relevant concept in medical decision-making, a concept also identified by Armstrong and Hilton [29] in relation to use of a urological diagnostic tool in secondary care. In the present study, it appears that whilst seeing the value in stratified care, the GPs wish to maintain the discretion to use their own skill and judgement where they feel appropriate, and in doing so to maintain a balance between ‘codified’ knowledge and ‘tacit’ knowledge.

It appears, then, that for GPs to feel that stratified care might be a useful addition to practice, it must be seen by them to enhance their existing skills and knowledge, i.e. the codified, systematised knowledge/information from the tool and recommended matched treatments needs to provide additional clinical knowledge. However, they must not feel they are losing a salient part of their professional identity: the freedom to exercise discretion, to ‘over-rule’ the tool and choose different treatment options for a patient when felt necessary. How patients perceive stratified care to affect GP discretion and decision-making will clearly have implications for how, they too, respond to its use in practice. Whilst it is intended that, in the future RCT testing stratified care, GPs will follow the matched treatment recommendations, there will be several treatment options for each patient subgroup, allowing GPs and patients to reach a shared decision. It is this balance between direction and discretion that must be emphasised to both GPs and patients to promote the use of stratified care in the RCT.

Another key finding was the extent to which stratified care could foster either agreement and increased patient engagement in treatment decisions, or conversely, disagreement and conflict, negatively impacting upon the therapeutic relationship. In relation to this theme, the theoretical domain of *professional role and identity* was again identified as salient, and specifically how this related to the perceived *goals* of the consultation.

Concerns expressed about the use of the prognostic tool impeding upon GP-patient rapport-building appeared to reflect some GPs’ aversion to the artificiality of asking patients questions from a pre-defined list, which they saw as an unwelcome departure from the their usual way of talking to patients. Another identified barrier was the concern expressed by both GPs and patients that the matched treatment options could lead to restricted patient choice. For GPs, these concerns drew on a consumerist view of contemporary healthcare in which patients are more inclined to impose their own views and expect greater treatment choice; which may lead patients to reject the recommended matched treatments. This correlates with the findings of Lupton [32] who reported Australian GPs’ views that patients have become ‘more assertive and knowledgeable, and more willing to challenge doctors’ ([32], p 486). Whilst we found evidence of patients also expressing concerns about potential conflict, these do not fit with the consumerist notion of patients vying for greater choice, but instead portrayed GPs exercising their authority to make the final decision. This aligns more to Pilnick and Dingwall’s [33] argument that the notion of patients as consumers is over-emphasised, and in fact GP-patient power asymmetries remain inherent in consultations. For some patients, this asymmetry was seen to be problematic if it meant not receiving desired treatments. Whilst views of the contemporary clinician-patient relationship differed between GPs and patients, the importance placed on constructive dialogue and discussion, and concern about patient choice being restricted, were common, perhaps signalling similar beliefs about the primary goals of the consultation. Any threat to this relationship was seen as having the potential to undermine key elements of both the GP’s and patient’s respective identities.

However, for those GPs and patients who reported positive beliefs about the consequences of using stratified care, it was felt this new way of working could facilitate discussion and patient engagement, with GP and patient participating in constructive dialogue that can be seen to align to the principles of a patient-centred approach based on mutual decisions around management [34]. From this perspective stratified care was represented as being an acceptable addition to the consultation.

Thus, stratified care being seen as a useful addition to practice depends upon GPs and patients seeing it as enhancing their relationship, as opposed to fragmenting it either through GPs perceiving that patients will disagree with recommended treatments, or through patients’ concerns about restricted access to desired treatments.

Another key finding related to the reported predominance of a primarily biomedical approach in GP consultations. For some GPs, a focus first on biomedical priorities reflects the organisational set-up of general practice, and beyond that, generalised views about the role of medicine. Western medicine has always prioritised biomedical thinking – reflecting Mishler’s [35] famous dichotomy between the ‘voice of medicine’ and the ‘voice of the lifeworld’. The former centres on diagnosis, which is commonly afforded a privileged status as being what Jutel and Nettleton ([5], p.739) term ‘the foundation of medical authority’. This privileged status was reflected in the data; with evidence suggesting some GPs perceive their primary professional role being as a diagnostician; a role supported by some patients’ apparent desire for a ‘quick fix’ through scans and surgical intervention.

Another factor driving a biomedical focus was a sense of fear amongst some GPs, with regard to overlooking serious, albeit rare, pathologies. Our data suggest that such fear may be driven in part by contemporary changes in general practice, whereby GPs face greater scrutiny and accountability, which Nettleton et al. ([25], p.333), drawing on the earlier work of Power [36], refer to as the ‘contemporary thrust of audit and regulation’. More than this, the data also suggest a professional fear of lasting damage to the therapeutic relationship and a personal fear of reputational damage rooted in a lack of confidence in the assessment and management of patients with musculoskeletal conditions. Such multi-level fear may result in an over-preoccupation with missing serious pathologies such as cancer. This finding reflects literature on how personal fear of patient complaints and risk of litigation has led clinicians to adopt a more cautious approach in their way of practising [25, 29].

In relation to the TDF, these findings were represented through a proposed relationship in which the domains of *environmental context and resources*, *professional role and identity*, *beliefs about capabilities*, and *emotions: fear* could all potentially influence GPs’ and patients’ perceived *goals* of the consultation as centring on biomedical priorities. Whilst stratified care is intended to supplement examination and diagnosis, strict adherence to a biomedical approach─ whether through factors related to the environmental context or individual determinants─ could present a barrier to its use if GPs are less receptive to an approach based on prognostic factors.

Despite the emphasis in the data on the role of the GP as a diagnostician, there was recognition that making a definitive diagnosis is often not achievable; this may be especially the case for many patients with musculoskeletal conditions, which can often be non-specific in nature [37]. This may have led some GPs to see the added benefit of the stratified care approach in allowing them to more confidently assess the patient’s likely prognosis. For GPs and patients to perceive stratified care as an acceptable addition to usual care in future, it will be important to highlight how it can add to the existing biomedical, diagnostic approaches, as well as emphasise its potential to improve GPs’ confidence by providing prognostic information in the face of diagnostic uncertainty.

The practical issue of embedding clinical tools to support decision-making within the short timeframe of the GP consultation, coupled with the concern that tools will not fit ‘naturally’, are frequently cited in the literature [10, 12, 13], and were again apparent in our data. As with the other identified themes, the relationship between the theoretical domains of *environmental context and resources*, and *goals* was identified as salient. For some GP participants, the impact of clinical tools in general can alter the structure of the consultation, impede upon their usual consulting style, and distract from the goal of assessing the patient; a view which resulted in *pessimism* about the use of stratified care. Again, however, the variation observed in the data suggests an increasing acceptance of the use of clinical tools on the part of at least some GPs, who indicated that their common use in general practice has led to them appearing more naturalised, rather than intrusions on the consultation.

Despite these contrasting perceptions, all the GP participants stressed that above all the process of completing the prognostic tool and accessing the recommended matched treatment options must not be time-consuming, a view reflected in the patient data. These views can again be seen to reflect the contemporary nature of general practice ─ the increased pressure on GPs to incorporate ever more protocols and measures into a limited timeframe – 10 min in the UK context – in the face of greater scrutiny and accountability, means that the GP’s time is a precious commodity. However, participants emphasised that the key to the use of stratified care was that it be seen to be clinically useful, and if this is the case then GPs would make time for it regardless of the time constraints.

Another factor cited as crucial for stratified care to be seen as an acceptable and useful addition to practice was that recommended matched treatment options involving onward referral to secondary care must be aligned with available services. GPs highlighted the futility of treatment options matched to the patient’s prognostic risk that are unavailable in their locality, or involve very lengthy waiting times. GPs and patients seeing stratified care as a useful addition to practice in future will therefore be contingent both upon recognition of its potential for added value, as well as whether it can be incorporated seamlessly into the existing structure of the consultation and aligned with local service provision.

### Strengths and limitations

A limitation of this study is that views were sought prior to the final development of the prognostic tool and matched treatments; discussions in focus groups/ interviews were therefore limited to the general principles of stratified care as opposed to the specifics of the intervention. However, this did allow for the findings to be incorporated into the refinement of the intervention, thus representing an advantage of the timing of the study. An additional limitation is that exploring views of the anticipated impact of the approach on clinical practice rather than participants’ actual experiences can be problematic, as this involves speculating about hypothetical barriers and facilitators to its use. Observational research to be carried out during the RCT will generate further insights into how stratified care is actually used and experienced in general practice.

Though attempts were made during focus groups/ interviews to encourage participants to discuss issues that they saw to be salient, the influence of the researchers’ contributions on participants’ discussions must be acknowledged; giving due consideration to their subjective views/beliefs. In particular, perhaps, the fact that the researchers in this study are closely involved with the development of the stratified care intervention, could have had the potential to influence the way in which participants’ views were elicited. However, the role of the researchers’ own subjectivities in the research process need not represent a limitation, but can be seen as an inevitable and integral part of that process [38]. In any case, the researchers made a conscious effort where possible not to impose their own priorities on the data-collection, and the variation in views observed suggests that participants were not led into adopting a particular stance in line with that of the researchers.

A strength of the study is the parallel investigation of both GPs’ and patients’ views. This is uncommon in the behaviour change literature using the TDF, and these dual perspectives add robustness to the conclusions drawn. The critical approach to using the TDF – in which relationships were identified between theoretical domains within themes developed through an initial thematic analysis – also represents a strength of the study.

## Conclusions

The theoretical domains of *knowledge*, *skills, professional role and identity, environmental context and resources*, and *goals* were identified as being particularly salient to GPs’ and patients’ perceptions of the acceptability, and anticipated barriers and facilitators to the use of stratified care. Views across the dataset were mixed; some participants signalling a lack of acceptability partly due to the perception that stratified care could undermine clinical autonomy; whilst others saw it as an acceptable addition to clinical decision-making, when supplemented by existing clinical judgement. Therefore, whilst a strong theme within the existing social science and healthcare intervention literature identifies clinicians wishing to maintain clinical autonomy through resistance to the ‘prioritisation of codified knowledge’ [27], our analysis using the TDF suggests that the picture may be more complex; for some GPs autonomy did not signal a rejection of clinical tools to support decision-making, but a desire to retain appropriate levels of discretion in following their recommendations. The importance given by GPs and patients to patient choice and engagement in treatment decisions was highlighted in the analysis, and the perceived effect of stratified care as either strengthening the therapeutic relationship or as being detrimental to it also contributed to views on its (lack of) acceptability. Findings also highlighted potential barriers to the use of stratified care relating to the increasing accountability, scrutiny and pressure on GPs in contemporary practice, particularly related to time-constraints of the consultation; however, GPs’ increased familiarity with using clinical decision-aids for other conditions represented a facilitator to its adoption.

Based on these findings, in order for GPs and patients to see stratified care as a potentially useful addition in primary care, it must be perceived to add to existing clinical *knowledge* and *skills*, whilst not undermining GPs’ and patients’ respective *identities and roles* and be seamlessly integrated into the *environmental context* of the general practice consultation. These findings have practical implications, particularly in refining the intervention and informing the content and methods of our clinician support packages for GPs participating in the future RCT.

Findings also contribute to the theoretical debate on how best to encourage clinical behaviour change, and the possible role of the TDF in that process. Scrutinising the interrelated nature of the theoretical domains in the TDF and how these manifested in the data allowed for an in-depth, nuanced examination of GPs’ and patients’ views. This extended the use of the TDF outlined in previous literature, and provides a strong evidence-base from which to target key behaviour change determinants in supporting GPs in changing their clinical behaviour.

## Appendix

**Table 1:** Patient participant characteristics

Participant number	Age	Sex	Pain site	Pain duration	Pain severity (Rated A-E; A = mild; E = severe)	Occupation
1	45	F	Back	6–9 m	C	Office manager
2	64	M	Multi-site	2–3w	A	Retired
3	58	M	Back	1 yr<	D	Unemployed
4	59	M	Knee	4–5w	C	Communications manager
5	85	F	Shoulder	3–6 m	C	Retired
6	61	F	Multi-site	1 yr<	D	Case support worker
7	62	F	Knee	3–6 m	B	Nursing sister
8	67	M	Multi-site	6–9 m	A	Retired
9	49	M	Shoulder	0–1w	A	Lender offer chase team member
10	71	F	Shoulder	1 yr<	D	Not reported
11	64	F	Multi-site	1 yr<	D	Nurse manager
12	55	F	Neck	1 yr<	C	Not reported
13	51	F	Multi-site	9–12 m	E	Part time receptionist
14	48	F	Back	9–12 m	C	Teacher
15	51	M	Back	1 yr<	D	Mechanic
16	62	F	Multi-site	1 yr<	C	Personal Assistant
17	44	M	Back	2–3w	D	Not reported
18	66	F	Knee	1 yr<	E	Retired
19	79	F	Neck	6–8w	B	Retired
20	22	F	Knee	1 yr<	C	Catering assistant

## Additional files

Additional file 1: Focus Group Topic Guide: Patients. (DOCX 46 kb) Additional file 2: Focus Group Topic Guide: GPs. (DOCX 55 kb)

## Abbreviations

GP: General Practitioner
RCT: Randomised controlled trial
TDF: Theoretical Domains Framework
