# Supplementary material for: General Practitioners’ and patients’ perceptions towards stratified care: a theory informed investigation
Source: BMC Fam Pract. 2016 Aug 31;17(1):125. doi: 10.1186/s12875-016-0511-2 (PMC5007841; doi:10.1186/s12875-016-0511-2)
Supplement: Additional file 1: — Focus Group Topic Guide: Patients. (DOCX 46 kb) [file 12875_2016_511_MOESM1_ESM.docx]

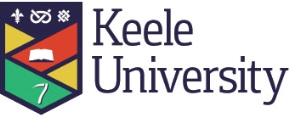

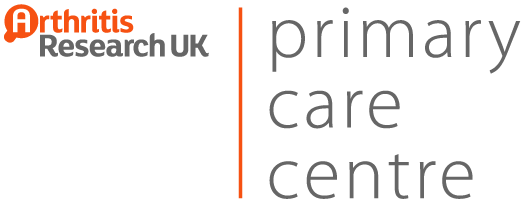


KAPS Focus Group Topic Guide: Patients

1. **Introduction**
   1. Brief overall introduction/thank you for coming
   2. Keele introductions followed by participant introductions
   3. Explain usual arrangements for: consent, recording, anonymity, focus group rules etc.
   4. Presentation of stratified care by the research team – outlining the rationale and benefits of stratified care, followed by questions from participants [10 mins ] *[This presentation will be a lay version of the presentation delivered to the GP focus groups]*

NB: additional prompts throughout around implications for:

Knowledge; Skills; Capabilities

Identity; Optimism; Pessimism

Beliefs about consequences

Reinforcements, rewards, incentives, consequences

Intentions; Goals

Memory, attention, decision making process

Social influences

Emotions

Self-monitoring

1. **Plenary discussion**
2. How satisfied are you with the care you have received or are currently receiving for your pain condition?
3. Would you change any aspect of care that you have received?
   - 1. Why/How?
4. How do you perceive patient care being informed by an approach that subgroups patients and matches these subgroups to treatments?
5. Do you think such an approach would be appropriate for your condition? Why/explain?
6. How might GPs explain to patients why they are using the tool? How might they explain the matching of treatments to the patient’s subgroup?
7. How should the tool be used in your opinion, e.g. self-completion by patients or by GP in face to face discussion with patients? Or separately?
8. Do you perceive any difficulties of a treatment matching approach?
9. **Close of discussion**
   1. Any other final remarks/additional views.
   2. Summary of discussion
   3. Reimbursement of travel expenses etc (where appropriate).
