# Supplementary material for: General Practitioners’ and patients’ perceptions towards stratified care: a theory informed investigation
Source: BMC Fam Pract. 2016 Aug 31;17(1):125. doi: 10.1186/s12875-016-0511-2 (PMC5007841; doi:10.1186/s12875-016-0511-2)
Supplement: Additional file 2: — Focus Group Topic Guide: GPs. (DOCX 55 kb) [file 12875_2016_511_MOESM2_ESM.docx]

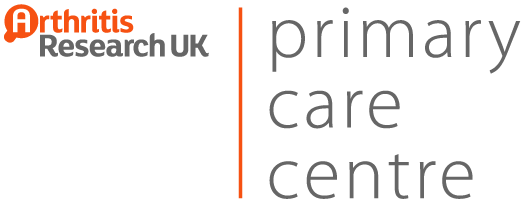

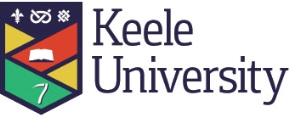


KAPS Focus Group Topic Guide: GPs

1. **Introduction**
   1. Brief overall introduction/thank you for coming
   2. Keele introductions followed by participant introductions (invite them to provide details on their length of time in primary care, any special interests, previous experience/background, experience of using STarTBack if any)
   3. Explain usual arrangements for: consent, recording, anonymity, focus group rules etc.
      1. NB: signing of consent form to be at termination of discussion as check that consent still in place.
   4. Presentation of stratified care by the research team – outlining the rationale and benefits of stratified care, followed by questions from participants. This presentation will explain that stratified care focuses on prognosis + complexity, i.e. statistical epidemiology alongside clinical insight/nous [7-10 mins ]

NB: additional prompts throughout around implications for:

Knowledge; Skills; Capabilities

Identity; Optimism; Pessimism

Beliefs about consequences

Reinforcements, rewards, incentives, consequences

Intentions; Goals

Memory, attention, decision making process

Social influences

Emotions

Self-monitoring

1. **Plenary discussion: general views on the acceptability of stratified care:** (where possible invite participants to expand on their responses and other members of the group to comment/express differing views)
   1. What are your initial views of using a stratified care approach in the case of patients with musculoskeletal conditions?

i. How does stratified care compare with your usual practice?

ii. Would a stratified care approach affect workload or relationships with other healthcare professionals in the care pathway?

1. What in your opinion might be the added value of stratified care compared to usual care, for the 5 most common musculoskeletal pain presentations?
2. What, if any, drawbacks might there be?
3. How do you envisage the tool informing decision-making and how might this sit alongside your own clinical judgement?
   1. What are your initial thoughts about classifying patients in this way – e.g. low, medium and high risk (of poor outcome) / complexity?
      1. What do you think about the validity and acceptability of making decisions about treatment based on patients’ risk of poor outcome?
      2. How might a focus on poor outcome sit alongside a diagnostic approach? – Are there any issues/tensions surrounding this?
      3. For which of the 5 sites of musculoskeletal conditions we are proposing would a stratified care approach be most/least beneficial?
4. **Adoption of Stratified Care in Clinical Practice**

**3.1 Use of the Screening Tool:**

1. How might the ‘stratified care **tool**’ be used in consultations with patients?
2. Are there any issues around the fact that the GP would need to input information into the computer during the consultation?
   1. Would this represent a change to your own consulting style?
   2. If so, what would help you to change this in order to accommodate the use of the tool?
   3. How do you think patients might respond to the GP using the computer during consultations?
3. How can GPs communicate risk information to patients?
4. What are possible effects on the consultation dynamic, i.e. GP-patient relationship?

**3.2 Matched Treatment Options:**

- 1. Do you feel that having recommended treatments matched to prognostic risk would benefit your clinical decision-making?
  2. What sort of characteristics might a patient have that would lead you to consider them to be:
     1. low risk/complexity
     2. medium risk/complexity
     3. high risk/complexity

d. How might a stratified care approach impact upon the treatment of ‘low risk’ patients?

- 1. What sorts of resources would be useful for treatment of low risk patients?

1. What treatments do you feel are most appropriate for treating medium and high risk patients?
2. Are there resource issues that need to be considered? If so, what?
3. What, in your opinion, are the potential barriers and facilitators to using a stratified care approach?
4. What resources would help you to use stratified care? What additional training/skills might be needed?
5. **Close of discussion** (5 mins)
   1. Any other final remarks/additional views.
   2. Summary of discussion
   3. Signing of consent form as a check that consent is still in place.
   4. Check if participants would like to be updated on the progress of the study and/or receive a summary of the findings.
   5. Reimbursement of travel expenses etc (where appropriate)
